# Awareness and Knowledge of Exercise Physiology Among Syrian Medical Students: A Cross‐Sectional Study

**DOI:** 10.1155/bmri/9148602

**Published:** 2026-04-07

**Authors:** Fater A. Khadour, Younes A. Khadour

**Affiliations:** ^1^ Department of Rehabilitation, Faculty of Medicine, Homs University, Homs, Syria; ^2^ Department of Rehabilitation, Tongji Hospital, Tongji Medical College, Huazhong University of Science and Technology, Wuhan, Hubei, China, hust.edu.cn; ^3^ Department of Physical Therapy, Cairo University, Cairo, Egypt, cu.edu.eg

**Keywords:** exercise, medical students, physical activity, physiology, Syria

## Abstract

**Background:**

Exercise physiology is a specialized field of physiology that focuses on examining the effects of exercise and physical activity on the structure and function of living organisms. Knowledge of exercise physiology is essential for enhancing athletic performance, enabling individuals to adapt to extreme conditions, and managing chronic diseases. The objective of this research was to assess the level of knowledge in exercise physiology among medical students in Syria, who are future healthcare professionals.

**Methods:**

The data was collected from a cohort of 704 undergraduate medical students who were actively following clinical internships. To evaluate their understanding of exercise physiology, a web‐based survey was utilized as a research tool. The survey is aimed at assessing participants′ foundational knowledge about diverse physiological concepts linked to physical exercise, encompassing topics such as respiratory exchange ratio, physical fitness, V̇O_2_ max (maximum oxygen uptake), and exercise at high altitudes.

**Results:**

A total of 55.5% of the participants exhibited a poor level of knowledge in exercise physiology. Misconceptions were evident regarding various aspects, including the definition (75.7%), application (64.4%), significance (55.3%), and related physiological factors (58.2%) of V̇O_2_ max. Furthermore, misconceptions were identified in the assessment of physical fitness, with proportions of 51.6%, 42.6%, and 38.8%, respectively, for specific evaluation questions. Similar misconceptions were prevalent in areas such as muscle groups and energy systems during training (34.9%), training prescription variables (42.8%), respiratory exchange ratio (64.4%), and high‐altitude performance (69.9%). Additionally, statistically significant differences in knowledge levels were observed based on respondent characteristics (*p* < 0.05).

**Conclusions:**

Syrian medical students demonstrated limited knowledge and moderate awareness of exercise physiology. These findings emphasize the need to enhance medical curricula by integrating exercise physiology more effectively and promoting active, applied learning strategies to strengthen future physicians′ competence in exercise‐based patient care.

## 1. Introduction

Physiology is the scientific study of how living organisms function, encompassing the roles of biomolecules, cells, tissues, and organ systems, as well as how these processes respond to environmental stimuli. Within this field, exercise physiology is a specialized branch that focuses on how physical activity and training influence the body′s structure and function [1]. Over time, research in exercise science has evolved from basic observations to multidisciplinary studies integrating physiology, biochemistry, and molecular biology [2].

Physical activity, defined as any skeletal muscle movement requiring energy expenditure in the form of adenosine triphosphate (ATP), is vital for health maintenance and disease prevention [3]. Exercise, a structured and repetitive form of physical activity, enhances physical fitness and supports the function of major systems such as the cardiovascular, respiratory, and musculoskeletal systems [4]. Regular participation in physical exercise improves physiological function, enhances overall health, and reduces the prevalence of chronic diseases such as cardiovascular disease, hypertension, cancer, and diabetes [5, 6]. Moreover, while moderate‐intensity exercise strengthens immune function, excessive or intense exercise may temporarily suppress it [7].

Exercise physiology provides scientific insight into these adaptations and underpins evidence‐based recommendations for health promotion and disease prevention [8]. Physicians play a crucial role in influencing patients′ health behaviors by promoting physical activity, but their effectiveness often depends on both awareness and knowledge [9, 10]. Awareness represents an individual′s recognition and understanding of the relevance and importance of exercise physiology within medicine. It is the first stage of cognitive engagement that precedes knowledge acquisition and practical application. Without adequate awareness, medical students may undervalue the significance of exercise science in disease prevention and treatment. Thus, enhancing awareness is essential for fostering deeper knowledge and encouraging the integration of exercise principles into clinical practice [1, 5, 11].

Understanding exercise physiology is fundamental for comprehending its preventive and therapeutic roles in chronic diseases [1, 5, 11, 12]. It also contributes to optimizing athletic performance and adaptation to environmental extremes such as high altitude [13]. However, persistent misconceptions about exercise physiology among healthcare professionals can hinder safe and effective exercise prescription, underscoring the importance of education in this area [14].

A strong grasp of exercise physiology not only improves clinical competence in prescribing exercise but also enriches medical students′ understanding of normal and pathological physiology. This includes insights into how physical activity influences cardiovascular and metabolic health and its relationship with conditions such as hypertension and coronary artery disease. Awareness and knowledge of exercise physiology together empower future physicians to guide patients toward healthier lifestyles and provide informed exercise‐related counseling.

Previous studies have identified misconceptions about physiological concepts among nursing, biology, and sports science undergraduates in the United States and the United Kingdom [15, 16]. Despite the well‐established role of exercise in promoting health and preventing chronic disease [17], there is limited research assessing both the awareness and knowledge of exercise physiology among medical students, particularly in Syria. Therefore, this study is aimed at evaluating the awareness and knowledge of exercise physiology among Syrian medical students, identifying educational gaps to support future curriculum enhancement.

## 2. Methods

### 2.1. Participants

This descriptive cross‐sectional survey was carried out in Syria from January 2024 to March 2024. Students who were suitable for the study included (1) undergraduate medical students in their clinical internship training phase, specifically those in the fourth, fifth, and sixth years of their medical education, and (2) those who are at least 18 years old. Those younger than 18 years or not currently undergoing clinical internship training as undergraduate medical students were excluded from the study.

The approximate sample size for this study was computed using the Raosoft [18] (sample size calculator), with a 5% margin of error, a confidence interval of 95%, and a distribution of responses equal to 50%.

The number of undergraduate medical students participating in clinical internships in Syria is estimated to be about 12,550. The minimum sample size calculated was 540 participants; however, the current study had almost 700 students. This study was approved by the Al‐Baath University Institutional Review Board (IRB) Committee (No. 043287654).

### 2.2. Study Design

This survey study was carried out in Arabic and comprised multiple parts. Part A presented a concise overview of the study′s topic and objectives, emphasizing that participation was voluntary and anonymous. It also comprises a consent form for participants to agree to participate in the research and allow the publication of the results.

Part B of the survey focused on gathering sociodemographic information about the participants; this included age, sex, marital status, and place of residence. This part also explored participants′ beliefs and attitudes toward physical exercise.

Moreover, Part B is aimed at investigating variables that could potentially impact the participants′ knowledge and academic achievement in exercise physiology. These variables encompassed the participants′ approach to preparing for medical exams during their essential medical sciences years.

Part C of the survey was adapted from a study by Leal et al. [19]. This part included 11 items previously developed to assess participants′ basic understanding of exercise physiology. This part is aimed at evaluating the participants′ knowledge of various physiological concepts related to physical exercise. These concepts included the respiratory exchange ratio (RER), physical fitness, maximal oxygen consumption (V̇O_2_ max), and their practical applications.

Firstly, the survey was written in English, then translated into Arabic by language specialists, and then retranslated into English to ensure consistency in the meaning of terms and concepts (before collecting the study sample). A pilot study was done on 40 students, and the questionnaire was revised by the principal statistician. The tool′s reliability in assessing acceptance was evaluated using Cronbach′s alpha, which was used to determine the “internal consistency” of the questionnaire and showed an acceptable score of 0.88.

### 2.3. Measurement of Awareness

Awareness was operationally defined as the recognition and perceived importance of exercise physiology in clinical education and patient care. To measure it, specific items were included in Part B of the questionnaire, asking students to indicate their level of agreement on a 5‐point Likert scale (1 = *strongly disagree*, 5 = *strongly agree*) with the following statements:

1. “I am familiar with the field of exercise physiology.”
2. “I believe exercise physiology is important for medical education.”
3. “Doctors should be aware of exercise physiology principles when managing patients.”
4. “Our medical curriculum adequately covers exercise physiology topics.”

Responses were combined to generate an overall awareness score, with higher mean values indicating greater awareness. Based on the mean and standard deviation, awareness levels were categorized as low (≤ 2.99), moderate (3.00–3.99), and high (≥ 4.00). Cronbach′s alpha for the awareness subscale was 0.86, demonstrating good internal consistency.

### 2.4. Data Collection Procedure

A web‐based survey was employed to collect the data, and Google Forms was used to create the online questionnaire. The questionnaire was distributed to the targeted participants, who were medical students during their clinical internships, through various teaching platforms specifically designed for medical students. Additionally, social media platforms such as WhatsApp were utilized to reach a wider audience of participants.

Participants completed the questionnaire independently without prior preparation or access to study materials. No review materials were provided before participation to ensure that responses accurately reflected existing knowledge and awareness levels.

## 3. Statistical Analysis

A scoring system was implemented to evaluate the students′ knowledge of physiology. Each question in Part C of the questionnaire had one correct answer, and participants received a score of 1 for a proper response, 0 for an incorrect response, or 0 for selecting “I do not know.” The student scores for each participant were then converted into percentages, ranging from 0% to 100%. These knowledge percentages were subsequently categorized into three groups based on the scoring system: a poor knowledge category for scores of 60% or below, a moderate knowledge category for scores between 60.01% and 80%, and a good knowledge category for scores of 80.01% or above [20].

Descriptive statistics, such as frequency (*N*) and percentages (%), were used to summarize the collected data. The chi‐square (*χ* ^2^) test was employed to explore the relationships between the participants′ knowledge levels and various factors such as sociodemographic characteristics, beliefs toward physical exercise, and predictors of academic achievement. A *p* value of ≤ 0.05 was considered statistically significant. The statistical analysis was conducted using Version 26 of the SPSS software by IBM Corporation.

## 4. Results

The sociodemographic details of the study′s participants are shown in Table 1. This study involved 704 medical students enrolled in undergraduate clinical internships. The participants′ average age was 21.26 years. About 50% of the participants were male, and more than 95% were single. Most of the participants, 521 (74.0%), were urban residents.

**Table 1:** Sociodemographic beliefs toward physical exercise and predictors of academic achievement of the study participants.

Variable	*M* *e* *a* *n* ± *S* *D* or *N* (%)
Age (years)	21.26 ± 2.11
Sex	
Male	329 (46.7%)
Female	375 (53.3%)
Marital status	
Single	673 (95.6%)
Married	31 (4.4%)
Place of residence	
City	521 (74.0%)
Village	183 (25.0%)
Curriculum year	
4th year	231 (32.9%)
5th year	307 (43.7%)
6th year	166 (23.4%)
Rotation when participating in the study	
Internal medicine	180 (25.6%)
Surgery	211 (29.9%)
Pediatrics	79 (11.2%)
Obstetrics/gynecology	61 (8.7%)
Emergency room	31 (4.4%)
Others	142 (20.2%)
How well did you prepare for your medical exams during the basic medical sciences years (Years 1–3)?	
Poor	41 (5.8%)
Fair	79 (11.2%)
Good	191 (27.1%)
Very good	262 (37.2%)
Excellent	131 (18.7%)
Have you ever failed any medical course during your study in the basic medical years (Years 1–3)?	
Yes	470 (66.8%)
No	234 (33.2%)
Cumulative GPA in all basic medical years	
Poor	2 (0.3%)
Fair	31 (4.4%)
Good	183 (26.1%)
Very good	379 (53.8%)
Excellent	109 (15.4%)
What is the kind of hospitals do you get more knowledge about medical sciences during your clinical training?	
University hospitals mainly	421 (59.8%)
Ministry of Health hospitals mainly	153 (21.7%)
Private medical services hospitals mainly	35 (4.8%)
More than one of the above	95 (13.7%)
I routinely (3–5 days/week) exercise as a part of my lifestyle to maintain my health	
Yes	295 (41.9%)
No	409 (58.1%)
I believe that it is important for medical doctors to counsel patients about exercise	
Strongly disagree	7 (0.9%)
Disagree	15 (2.1%)
Neutral	140 (19.9%)
Agree	349 (49.6%)
Strongly agree	193 (27.5%)
I believe that I have sufficient knowledge to counsel a patient on exercise prescription in the future	
Strongly disagree	29 (4.1%)
Disagree	131 (18.6%)
Neutral	213 (30.2%)
Agree	245 (34.8%)
Strongly agree	86 (12.3%)
I believe that the MD curriculum should include more education on exercise physiology and prescription	
Strongly disagree	6 (0.8%)
Disagree	28 (4.1%)
Neutral	153 (21.7%)
Agree	321 (45.6%)
Strongly agree	196 (27.8%)

There were 231 (32.9%) fourth‐year medical students, 307 (43.7%) fifth‐year medical students, and 166 (23.4%) sixth‐year medical students. The distribution of participants in clinical internships at the time of the study is as follows: 180 (25.6%) students in internal medicine, 211 (29.9%) students in surgery, 79 (11.2%) students in pediatrics, 61 (8.7%) students in obstetrics and gynecology, 31 (4.4%) students in the emergency room (ER), and 142 (20.2%) students in other clinical rotations.

The MD students were requested to reflect on their study habits over the first 3 years of their education, with a particular emphasis on their preparation for MD tests and academic achievement. A minority of MD students, 120 (17.0%), who participated in the survey expressed fair to poor preparation, whereas the majority, 584 (83%), stated that they studied in a very good to excellent manner for their MD exams.

Furthermore, a significant proportion of participants, 470 (66.8%), did not experience any failures in their medical courses during the initial 3 years of their MD study. Furthermore, almost 671 (95.3%) of the students had cumulative GPAs ranging from good to excellent in all of the foundational medical subjects. Concerning the participants′ perspectives on physical activity knowledge, most students recognized the importance of doctors providing exercise advice to patients. They believed that the MD curriculum needed to include more exercise physiology coursework.

The knowledge of exercise physiology survey comprised 11 items designed to assess participants′ familiarity with basic concepts in exercise physiology. There were five potential answers (a–e) for each item. Table 2 represents the answers provided by the medical students to this survey. In Item 1, a significant majority of 535 (74.7%) of the participants could not provide a correct definition of V̇O_2_ max. Regarding Item 2, 453 (64.4%) of participants had misconceptions regarding using V̇O_2_ max in physical examination. In Item 3, 410 (58.2%) of participants were unfamiliar with the physiological elements that affect V̇O_2_ max. Additionally, in Item 4, 390 (55.3%) of the participants did not comprehend the significance of V̇O_2_ max in the prescription of exercise.

**Table 2:** Summary of exercise physiology knowledge questionnaire responses.

**Question**	**N (%)**
1. The Cardiopulmonary Exercise Stress Test evaluates the maximal oxygen uptake (V̇O_2_ max) and the ventilatory anaerobic threshold. Consider the alternatives below and mark the correct alternative:	254 (34.8%)
(a) V̇O_2_ max may be defined as the maximum oxygen uptake capacity, transport and use during dynamic exercise involving large body muscle mass	94 (13.4%)
(b) V̇O_2_ max may be defined as the maximum volume of oxygen that can enter and leave the lungs during dynamic exercise involving large body muscle mass	103 (14.6%)
(c) V̇O_2_ max may be defined as the maximum volume of oxygen needed to perform an exercise as quickly as possible	74 (11.5%)
(d) V̇O_2_ max may be defined as the maximum oxygen uptake capacity, transport and use during dynamic exercise sustained during a stable phase involving large body muscle^a^	169 (24.3%)
(e) I do not know	10 (1.4%)
2. With respect to maximal oxygen uptake (V̇O_2_ max), answer the correct alternative	
I. May be used for the prescription of training with aerobic characteristics	
II. May be used to verify the effectiveness of a training plan and/or medical treatment	
III. May be used to verify which physiological system limits maximum exercise	
(a) Only proposition I is correct	64 (9.1%)
(b) Only propositions I and II are correct	75 (10.6%)
(c) Only propositions II and III are correct	96 (13.6%)
(d) All propositions are correct^a^	251 (35.6%)
(e) I do not know	218 (31.1%)
3. The physiological factors that influence maximal oxygen uptake (V̇O_2_ max) are	
(a) Release of oxygen to the muscle	104 (14.7%)
(b) Oxygen uptake by muscle	111 (15.8%)
(c) Genetic and physical training	51 (7.2%)
(d) All alternatives are correct^a^	294 (41.8%)
(e) I do not know	144 (20.5%)
4. Maximal oxygen uptake (V̇O_2_ max)	
(a) May be used to predict aerobic performance	50 (7.2%)
(b) Is a cardiovascular fitness index	94 (13.4%)
(c) May be used to prescribe aerobic exercise	41 (5.8%)
(d) All the above alternatives are correct^a^	314 (44.7%)
(e) I do not know	204 (28.9%)
5. For a laboratory test to be effective, several key factors must be considered. Which of the following is/are true?	
(a) Laboratory tests must be repeated regularly	151 (21.4%)
(b) Laboratory tests must be valid and reliable	95 (13,5%)
(c) Laboratory tests must be the most specific available for the sport	43 (6.1%)
(d) All alternatives are correct^a^	341 (48.4%)
(e) I do not know	74 (10.6%)
6. Tests of cardiorespiratory fitness are used to	
(a) Determine the level of cardiorespiratory fitness and set goals for fitness programs as well as prescribe safe and effective physical exercise	91 (12.9%)
(b) Detect improvements in cardiorespiratory fitness as a result of physical training	64 (9.1%)
(c) Provide information in relation to health status	31 (4.4%)
(d) All alternatives are correct^a^	404 (57.4%)
(e) I do not know	114 (16.2%)
7. Regarding the importance of physical evaluation, analyse the alternatives below and mark the correct one	
(a) Physical evaluation outlines the physiological role of the subject and allows prescription of individualised training	92 (13.1%)
(b) Physical evaluation allows verification of the effectiveness of a training programme	107 (15.2%)
(c) Physical evaluation is useful only for athletes, because athletes train within the limits of physiological systems	15 (2.1%)
(d) Alternatives ‘a’ and ‘b’ are correct^a^	431 (61.2%)
(e) I do not know	59 (8.4%)
8. Training programmes must be specific by targeting	
(a) Muscle groups used in competition	87 (12.3%)
(b) Energy systems that provide fuel for competition	66 (9.3%)
(c) Muscle groups and energy systems not used during competition	21 (3.1%)
(d) Alternatives ‘a’ and ‘b’ are correct^a^	457 (65.1%)
(e) I do not know	73 (10.2%)
9. The variable(s) that constitute workload aspects of a physical training programme include(s)	
(a) Intensity	121 (17.2%)
(b) Duration	74 (10.5%)
(c) Frequency	81 (11.6%)
(d) All alternatives are correct^a^	403 (57.2%)
(e) I do not know	25 (3.5%)
10. A non‐invasive technique often used to estimate the contribution of the substrate to the production of adenosine triphosphate (ATP) is by measuring	
(a) Maximal oxygen uptake (V̇O_2_ max)	237 (33.5%)
(b) RER^a^	249 (35.4%)
(c) Lactate threshold	81 (11.5%)
(d) Anaerobic threshold	39 (5.7%)
(e) I do not know	98 (13.9%)
11. Physical performance at high altitudes may be affected by	
(a) Decreased difference in partial pressure of oxygen between alveolar air and venous blood from the pulmonary capillary^a^	210 (30.1%)
(b) Increased percentage O_2_ inspired	117 (16.5%)
(c) Increased haemoglobin saturation	71 (10.1%)
(d) Decreased percentage O_2_ inspired	152 (21.6%)
(e) I do not know	154 (21.7%)

^a^Indicates correct answer.

Concerning Item 5, 363 (51.6%) of the participants stated misconceptions regarding the evaluation of physical fitness, while 300 (42.6%) and 273 (38.3%) had misconceptions in Items 6 and 7, respectively. Regarding Item 8, 247 (34.9%) of the participants were unaware that energy systems are used during physical exercise. For Item 9, which evaluated knowledge of elements related to training prescriptions, 301 (42.8%) of the participants provided incorrect answers. Only 249 (35.4%) of the participants answered Item 10 correctly, demonstrating their knowledge of RER. Lastly, in Item 11, which examined participants′ understanding of physical performance in high‐altitude environments, 494 (69.9%) reported misunderstandings.

The students′ mean knowledge percentage was lower than 50%; among them, the majority (55.5%) had poor knowledge, followed by moderate knowledge (25.4%), and good knowledge (19.1%), as shown in Table 3.

**Table 3:** Profile of MD students′ exercise physiology knowledge categories.

Variable	*M* *e* *a* *n* ± *S* *D*	Knowledge category (%)		
		Poor	Moderate	Good
Exercise physiology knowledge	47.6 ± 24.7	55.5	25.4	19.1

The results of this study revealed a substantial association between levels of knowledge and many participants′ characteristics. Table 4 presents the results of the chi‐square (*χ* ^2^) test. These characteristics include marriage status (*p* = 0.023), place of residence (*p* = 0.002), the curriculum year (*p* = 0.015), approach to exam preparation used during the fundamental medical sciences years (*p* = 0.005), and the kind of hospital where the majority of the students′ medical sciences education was received during internship period (*p* = 0.061).

**Table 4:** Chi‐square (*χ* ^2^) test of association between sociodemographic characteristics, academic information, beliefs toward physical exercise, and predictors of academic achievement with respect to exercise physiology knowledge category.

Demographic variable	Category	*N*	Exercise physiology knowledge category *N* (%)			*χ* ^2^	*p* value
			Poor	Moderate	Good		
Gender	Male	329	227 (69.1%)	53 (16.1%)	49 (14.8%)	2.437	0.743
	Female	375	241 (64.2%)	97 (25.9%)	37 (9.9%)		
Marital status	Single	673	571 (84.8%)	45 (6.7%)	8.5 (57%)	12.451	0.023
	Married	31	18 (58.1%)	4 (12.8%)	9 (29.1%)		
Place of residence	City	521	361 (69.3%)	111 (21.3%)	49 (9.4%)	17.582	0.002
	Village	183	83 (45.3%)	44 (24.1%)	56 (30.6%)		
Curriculum year	4th year	231	137 (59.3%)	26 (11.3%)	68 (29.4%)	12.624	0.015
	5th year	307	163 (53.1%)	110 (35.8%)	34 (11.1%)		
	6th year	166	123 (74.1%)	31 (18.7%)	12 (7.2%)		
Preparation for medical exams during the basic medical sciences years	Poor	41	28 (67.7%)	11 (25.2%)	3 (7.1%)	17.483	0.005
	Fair	79	43 (54.4%)	27 (34.2%)	9 (11.4%)		
	Good	191	107 (66.1%)	86 (45.1%)	21 (10.8%)		
	Very good	262	166 (63.3%)	79 (30.2%)	17 (6.5%)		
	Excellent	131	83 (63.4%)	41 (31.3%)	7 (5.3%)		
Failed any medical course during the basic medical years	Yes	234	133 (56.8%)	75 (32.1%)	26 (11.1%)	5.621	0.731
	No	470	321 (68.3%)	110 (23.4%)	39 (8.3%)		
Cumulative GPA in all basic medical years	Poor	2	1 (50.0%)	1 (50.0%)	0 (0%)	14.637	0.362
	Fair	31	20 (64.5%)	7 (22.6%)	4 (12.9%)		
	Good	183	125 (68.3%)	41 (22.4%)	17 (9.3%)		
	Very good	379	273 (72.1%)	81 (22.3%)	25 (6.6%)		
	Excellent	109	61 (56.1%)	34 (32.1%)	14 (12.7%)		
Rotation when participating in the study	Internal medicine	180	111 (61.7%)	47 (26.1%)	22 (12.2%)	11.473	0.361
	Surgery	211	124 (58.8%)	61 (28.9%)	26 (12.3%)		
	Pediatrics	79	47 (59.5%)	24 (30.4%)	8 (10.1%)		
	Obstetrics/gynecology	61	39 (63.9%)	16 (26.2%)	6 (9.9%)		
	Emergency room	31	18 (58.1%)	9 (29.1%)	4 (13.8%)		
	Others	142	85 (59.8%)	36 (25.4%)	21 (14.8%)		
What is the kind of hospitals do you get more knowledge about medical sciences during your clinical training?	University hospitals mainly	421	291 (69.1%)	113 (26.8%)	17 (4.1%)	11.942	0.061
	Ministry of Health hospitals mainly	153	92 (60.2%)	55 (35.9%)	6 (3.9%)		
	Private medical services hospitals mainly	35	23 (65.7%)	9 (25.7%)	3 (8.6%)		
	More than one of the above	95	64 (67.4%)	22 (23.1%)	9 (9.5%)		
I believe that it is important for medical doctors to counsel patients about exercise	Strongly disagree	7	4 (57.1%)	3 (42.9%)	0 (0%)	15.392	0.411
	Disagree	15	19 (60.1%)	4 (26.6%)	2 (13.3%)		
	Neutral	140	93 (66.4%)	30 (21.4%)	17 (22.2%)		
	Agree	349	274 (78.5%)	51 (14.6%)	24 (6.9%)		
	Strongly agree	193	147 (76.1%)	35 (18.1%)	11 (5.8%)		
I believe that I have sufficient knowledge to counsel a patient on exercise prescription in the future	Strongly disagree	29	17 (58.6%)	9 (31.1%)	3 (10.3%)	13.435	0.091
	Disagree	131	94 (71.7%)	25 (19.1%)	12 (9.2%)		
	Neutral	213	126 (59.1%)	55 (25.8%)	32 (15.1%)		
	Agree	245	149 (60.8%)	57 (23.3%)	39 (15.98%)		
	Strongly agree	86	56 (65.1%)	23 (26.7%)	7 (8.2%)		
I believe that the MD curriculum should include more education on exercise physiology and prescription	Strongly disagree	6	5 (83.3%)	1 (16.7%)	0 (0%)	7.019	0.516
	Disagree	28	17 (60.7%)	7 (25.1%)	4 (12.2%)		
	Neutral	153	113 (73.8%)	30 (19.6%)	10 (6.6%)		
	Agree	321	239 (74.4%)	68 (21.2%)	14 (2.4%)		
	Strongly agree	196	116 (59.2%)	59 (30.1%)	21 (10.7%)		

Married respondents represented a larger percentage of respondents in the good knowledge group than single respondents. Additionally, respondents who resided in villages had a higher proportion in the good knowledge category than those who lived in urban areas.

Students in their fifth and sixth years of MD studies reported being more knowledgeable than those in their fourth year of study. Furthermore, compared to students with lower test preparation levels, students who reported excellent preparation for medical exams during their foundational medical sciences years were more likely to be classified into the good knowledge group. Finally, a good level of knowledge was correlated with a greater proportion of participants who learned more about medical skills during their internships in university hospitals than those who received training in different kinds of hospitals.

### 4.1. Awareness of Exercise Physiology

To address the reviewer′s comment, the results now include a distinct section describing awareness findings. Table 5 summarizes the awareness results. Overall, 62.1% of students demonstrated moderate awareness, 23.3% showed high awareness, and 14.6% had low awareness. Awareness scores were significantly associated with the year of study (*p* = 0.019) and prior exposure to physical activity topics within the curriculum (*p* = 0.031). No significant differences were found by gender or residence.

**Table 5:** Awareness levels of exercise physiology among Syrian medical students.

Awareness level	Number of students (*N*)	Percentage (%)
Low	103	14.6
Moderate	437	62.1
High	164	23.3

*Note:* Awareness was measured using a 5‐point Likert scale (1 = *strongly disagree*, 5 = *strongly agree*) based on four items assessing familiarity with, perceived importance of, and curriculum coverage of exercise physiology. Awareness scores were categorized as low (≤ 2.99), moderate (3.00–3.99), and high (≥ 4.00).

Students in later clinical years displayed higher awareness levels, indicating that increased exposure to practical training and clinical experience enhances understanding of exercise physiology′s relevance. Despite this, the overall moderate awareness level suggests a need for curriculum enhancements and targeted educational strategies to improve both awareness and knowledge of exercise physiology among Syrian medical students.

## 5. Discussion

A strong understanding of exercise physiology is crucial for healthcare providers and individuals passionate about training exercises and health. It enables professionals to establish and develop appropriate exercise programs to enhance physical abilities and enhancing overall health. The study is aimed at assessing future Syrian medical professionals′ knowledge of exercise physiology. The results of the study showed that Syrian undergraduate MD students′ knowledge of exercise physiology was inadequate during their clinical internship; this indicates the presence of misunderstandings and insufficient knowledge among MD students about the basic concepts of exercise physiology.

Previous research focused on the expertise of healthcare professionals regarding prescribed exercise and recommendations for physical activity. These studies consistently highlight a deficiency in healthcare providers′ knowledge and counseling abilities related to physical activity [9, 21]. For instance, O′Brien et al. conducted a study in Ireland and reported that physicians′ attitudes, comprehension, and implementation of physical activity counseling were significantly deficient. The findings of this study showed a notable difference between graduate and undergraduate programs′ availability of instruction in physical activity counseling [9].

Another survey study was conducted in the United Kingdom that evaluated final‐year medical students′ knowledge level regarding physical activity and revealed that the participating students had insufficient knowledge of physical activity standards [21]. However, there is a scarcity of studies explicitly examining the knowledge level of clinicians and medical undergraduates about exercise physiology.

V̇O_2_ max serves as a measure of an individual′s ability to transport and utilize oxygen during physical activity. It is utilized to estimate cardiorespiratory fitness [22], with higher levels of V̇O_2_ max associated with increased life expectancy and better overall health and vice versa [23]. The results of the present study indicate that a majority (74.7%) of MD undergraduates provided incorrect definitions of V̇O_2_ max. A study performed by Leal et al. showed that approximately 30% of the participants had insufficient information regarding the variables affecting V̇O_2_ max, which is consistent with the current study′s findings [19].

Furthermore, our study revealed that 64.4% of the students had misconceptions about the application of V̇O_2_ max in physical evaluation, while 55.3% held misconceptions about its importance in exercise prescription. These findings are consistent with the results of the study conducted by Leal et al., which found that approximately 50% of the participants displayed insufficient knowledge regarding the application of V̇O_2_ max in physical evaluation, while around 44% lacked understanding of its importance in exercise prescription [19].

Misconceptions about the concept and implementation of V̇O_2_ max can have various negative consequences. For instance, not recognizing that V̇O_2_ max is a crucial parameter for assessing aerobic fitness levels and measuring the effectiveness of training may lead to alternative fitness evaluation measures that may not accurately assess an individual′s aerobic fitness compared to V̇O_2_ max [24, 25]. Insufficient knowledge regarding the testing procedures for V̇O_2_ max or the factors that influence it, such as age, genetics, gender, and cardiorespiratory function, can lead to inaccurate expectations or incorrect analysis of test findings. Moreover, insufficient comprehension of the significance and application of V̇O_2_ max in health assessments can result in inappropriate prescribing of exercise, which can have adverse effects, including a higher rate of injuries and reduced physical efficiency [14].

Based on the available literature, there is limited information for comparison regarding the prevalence of misconceptions regarding the assessment of physical performance among clinical MD students, apart from the study conducted by Leal et al. [19]. However, the findings of the present study indicate that the participants had more misconceptions about the assessment of physical performance than those reported in Leal et al. The difference between the findings of this study and the findings from Leal et al. [19] can be attributed to reasons such as the differences in medical school curricula between Brazil and Syria.

Differences among medical curricula likely contribute to the variations observed in students′ awareness and knowledge of exercise physiology. In many institutions, exercise physiology content is inconsistently integrated into medical programs. Some curricula introduce these concepts during preclinical years as part of general physiology, while others delay or omit them altogether. When exercise‐related principles are not reinforced during clinical years, students may fail to connect theoretical knowledge with patient care applications. Moreover, a lack of emphasis on exercise science within assessment frameworks can reduce students′ motivation to study this topic deeply.

Medical education exhibits global variation, wherein different institutions emphasize distinct components of the curriculum. These variations extend to the inclusion of exercise physiology content in the curricula, the timing of its introduction, and other country‐specific features that are unique to medical education. The assessment of an individual′s physical fitness is crucial for recognizing potential health risks and developing a customized exercise program that aligns with their specific fitness goals and needs [26].

However, misconceptions surrounding physical fitness evaluation can lead to incorrect exercise recommendations and potentially adverse consequences. Furthermore, a lack of understanding regarding the necessary components of reliable laboratory tests, as well as misconceptions about the objectives and execution of these assessments, can undermine their accuracy and reliability. As a result, inappropriate exercise recommendations and inadequate outcomes may occur.

The present study′s findings indicate that a significant proportion of MD students lack awareness regarding the significance of targeting both muscle groups and energy systems when designing exercise programs. Previous research has also highlighted the essentiality of incorporating exercises that focus on both body musculature and energy systems to develop effective fitness exercise programs that promote optimal performance and minimize the risk of injury [27–29].

Regarding the understanding of workload aspects (frequency, intensity, and duration of training) in a physical training program [30], a study conducted by O′Keefe et al. found that 41% of the participating medical students had incorrect knowledge regarding these components [31]. This lack of understanding can have negative consequences, as it may lead to the development of training programs that are inadequate or ineffective in achieving optimal results.

Our study findings presented that a majority of medical students exhibited a lack of knowledge regarding the RER. The RER is a physiological parameter utilized to evaluate the relative utilization of carbohydrates and fats as energy sources during exercise. The RER rises with higher exercise intensity, and a high RER indicates that carbohydrates are the main energy source of the body, whereas a low RER suggests that fats are the main fuel source [32].

Understanding how the human body uses energy during physical exercise is critical for clinicians to enhance patients′ training programs and modify their dietary requirements appropriately. In the case of preparing individuals for endurance activities like running long distances, it is vital to prioritize moderate‐intensity exercises that promote the utilization of lipids as the primary energy source. A solid understanding of this concept empowers healthcare professionals to offer expert guidance and counseling, enabling individuals to make suitable dietary adjustments. This may involve modifying their diet to incorporate a higher proportion of fats compared to carbohydrates. As a result, accurately comprehending RER‐related aspects is critical for building well‐designed training programs that particularly target different types of energy to match the metabolic demands of that type of exercise.

Exercising in high‐altitude environments can present several physical challenges, including lower temperatures, reduced atmospheric pressure, hypoxia (reduced oxygen availability), and increased solar radiation [32]. These environmental changes can adversely affect the body, such as a decrease in the skeletal size of muscles, which may lead to a corresponding reduction in body mass [32]. Nevertheless, recent research has demonstrated that training at high altitudes has several benefits for athletes and normal people. It can increase oxygen transport, enhance aerobic capacity, and stimulate the production of erythropoietin, a hormone that promotes the production of red blood cells [32]. These physiological adaptations can lead to improved athletic performance and overall fitness.

To optimize training conditions, athletes often employ a periodization strategy that includes residing at high elevations and exercising at lower elevations. By residing at high altitudes, athletes can expose themselves to the physiological stressors of reduced oxygen availability, which triggers beneficial adaptations. They then train at lower altitudes, where the oxygen availability is higher, allowing for more intense and productive training sessions [33].

In addition to the aforementioned considerations, clinicians might have to counsel people to protect their bodies against excessive ultraviolet (UV) exposure while training or living at high elevations. This can be done by recommending protective clothing and applying sunscreen to exposed skin. UV radiation levels tend to be higher at high altitudes due to a thinner atmosphere and increased reflection from snow or ice.

Furthermore, being aware of medical disorders associated with high elevations, such as cerebral or pulmonary edema and acute mountain sickness [34], is crucial for physicians. This knowledge allows them to anticipate, diagnose, and treat these conditions promptly, potentially saving lives. Understanding the signs and symptoms of such disorders and being familiar with appropriate treatment options ensures that physicians can provide adequate medical care.

Additionally, our data revealed that individuals who were married and resided in rural areas demonstrated a higher level of exercise physiology knowledge compared to those who were single and lived in urban areas. This may be attributed to several factors, including an increased sharing of knowledge within the context of a married relationship. Married individuals may have more opportunities to discuss and share knowledge about exercise and health with their partners, leading to a higher understanding of exercise physiology. Another factor that may contribute to the higher level of knowledge among participants residing in villages is the potential presence of community‐based programs or local health initiatives. In rural settings, close‐knit communities are often formed, facilitating the sharing and dissemination of health‐related information. This community support and engagement can increase villagers′ awareness and understanding of exercise physiology. In contrast, urban areas may have a more fragmented community structure, potentially limiting the dissemination of health‐related information.

The chi‐square analysis revealed significant relationships between students′ knowledge levels and several demographic and academic variables, including marital status, place of residence, curriculum year, exam preparation approach, and training hospital type. Married participants exhibited higher knowledge levels than single students, possibly due to greater health‐related discussions and mutual motivation toward healthier lifestyles within households. Students residing in rural areas showed higher knowledge scores compared to those in urban regions. This difference may reflect stronger community‐based health promotion initiatives or closer personal engagement with physical activity in rural settings. Conversely, urban students may face time constraints or fewer community‐based opportunities for learning about exercise. A positive association between academic year and knowledge level suggests that exposure to clinical practice enhances understanding of exercise physiology. As students progress, they gain more opportunities to connect theoretical concepts with patient care. Furthermore, students who reported excellent exam preparation during preclinical years achieved higher knowledge scores, highlighting the impact of strong foundational study habits on long‐term comprehension. Lastly, students trained primarily in university hospitals demonstrated better knowledge compared to those trained in other institutions, likely reflecting access to richer academic environments and interdisciplinary learning opportunities.

To enhance awareness and knowledge, educational institutions should integrate exercise physiology systematically throughout medical training. This may include incorporating short modules or workshops focused on the clinical application of exercise physiology, embedding physical activity counseling into primary care training, and fostering collaboration between medical, physiotherapy, and sports science departments. Additionally, active learning strategies such as simulation‐based teaching, case‐based discussions, and student‐led health promotion projects can enhance engagement and retention. Ensuring that assessment frameworks evaluate applied knowledge of exercise physiology would further encourage consistent learning.

Insufficient knowledge of exercise physiology can affect healthcare professionals prescribing exercise and counseling patients, so identifying areas of weakness in exercise physiology knowledge among students of medicine can help tailor educational interventions to improve their understanding. By incorporating targeted educational interventions into the curriculum, future physicians can better understand exercise physiology [35].

Exercise physiology is a multidisciplinary field, and cooperation between healthcare professionals such as physicians, physiotherapists, and exercise physiologists can lead to comprehensive and individualized patient care [36]. By working together, healthcare professionals can integrate exercise as a critical component in preventative strategies and treatment plans for various medical conditions.

## 6. Study Limitations

This study has several limitations that should be mentioned. Firstly, relying on self‐reported surveys introduces the possibility of respondents inaccurately understanding or responding to specific items owing to cognitive limitations. Secondly, participants may share their data relying on memory and reading to potential recall bias. Thirdly, the possibility of disparities in access to the online survey platform may introduce bias in the data collected. However, despite these limitations, self‐reported surveys remain a vital instrument in numerous research studies. Another limitation is the potential influence of recall bias. Some participants may have had difficulty recalling concepts from their preclinical coursework, which could have affected their responses. However, this bias was likely minimal since the questionnaire focused on fundamental physiological principles expected to remain familiar throughout medical training.

## 7. Conclusion

In conclusion, the findings of this study demonstrate that Syrian medical students exhibit limited knowledge and moderate awareness of exercise physiology. These results align with previous research, such as the study by Aolymat et al., which highlighted similar gaps in understanding among medical students in Jordan [37]. This underscores the need for curricular enhancement and a greater emphasis on exercise science education within medical programs. Integrating exercise physiology across both preclinical and clinical training, supported by active learning strategies and interprofessional collaboration, could improve understanding and application of exercise concepts in future medical practice. Addressing these educational gaps will contribute to producing more competent physicians capable of promoting exercise as a vital component of patient care.

## Author Contributions

Younes A. Khadour wrote the initial draft of the manuscript, provided language help, and critically revised the manuscript; Fater A. Khadour critically revised the manuscript and supervised the entire course of the study. Fater A. Khadour and Younes A. Khadour contributed equally to this work, so they will be chosen to have joint first authorship.

## Funding

No funding was received for this manuscript.

## Ethics Statement

The studies involving human participants were reviewed and approved by the Al‐Baath University Institutional Review Board (IRB) Committee (No. 043287654). All procedures were conducted under the ethical principles outlined in the 1964 Declaration of Helsinki and its subsequent revisions. All our methods were carried out under relevant guidelines and regulations. Participants were informed of the study′s purpose and procedures. It was all voluntary; no names were taken, so we provided anonymous data collection.

## Consent

Informed consent was obtained from all the participants.

## Conflicts of Interest

The authors declare no conflicts of interest.

## Data Availability

The data generated in this study are available from the corresponding author on reasonable request.
